# A targeted solution for estimating the cell-type composition of bulk samples

**DOI:** 10.1186/s12859-021-04385-0

**Published:** 2021-09-26

**Authors:** Edwin J. C. G. van den Oord, Lin Y. Xie, Charles J. Tran, Min Zhao, Karolina A. Aberg

**Affiliations:** Center for Biomarker Research and Precision Medicine (BPM), School of Pharmacy, Virginia Commonwealth University, 1112 East Clay Street, McGuire Hall, Room 217, P.O. Box 980533, Richmond, VA 23298 USA

**Keywords:** Cell-type proportions, Statistical deconvolution, Targeted analysis, Replication studies, DNA methylation, Transcription

## Abstract

**Background:**

To avoid false-positive findings and detect cell-type specific associations in methylation and transcription investigations with bulk samples, it is critical to know the proportions of the major cell-types.

**Results:**

We present a novel approach that allows for precise estimation of cell-type proportions using only a few highly informative methylation markers. The most reliable estimates were obtained with 17 amplicons (34 CpGs) using the MuSiC estimator, for which the average correlations between the estimated and the true cell-type proportions were 0.889. Furthermore, the estimates were not significantly different from the true values (*P* = 0.95) indicating that the estimator is unbiased and the standard deviation of the estimates further indicate high precision. Moreover, the overall variability of the estimates as measured by the Root Mean Squared Error (RMSE), which is a function of both bias and precision, was low (mean RMSE = 0.038). Taken together, these results indicate that the approach produced reliable estimates that are both unbiased and highly precise.

**Conclusion:**

This cost-effective approach for estimating cell-type proportions in bulk samples allows for enhanced targeted analysis, which in turn will minimize the risk of reporting false-positive findings and allowing for detection of cell-type specific associations. The approach is applicable across platforms and can be extended to assess cell-type proportions for various tissues.

**Supplementary Information:**

The online version contains supplementary material available at 10.1186/s12859-021-04385-0.

## Background

In methylation and transcription studies that involve bulk samples it is critical to account for cell-type heterogeneity [1]. That is, tissue samples from different individuals may show variation in their cell-type proportions and the specific cell-types may show distinct methylation/transcription profiles. In association studies with bulk samples this may result in false positive findings in the regions that show differences across cell-types if cell-type proportions are correlated with the investigated outcome. Another reason for the importance of knowing the cell-type composition of a tissue is that differently methylated regions and differently expressed transcripts may be challenging to detect in bulk tissue. This is because cell-type specific effects may be “diluted”, e.g., effects may be of opposite signs in different cell-types or involve low abundance cells [2–4]. However, if the cell-type compositions for each sample are available, statistical deconvolution methods can be used to study cell-type specific associations. This statistical deconvolution approach was first introduced about 20 years ago [3] and has successfully been used for both transcription and methylation studies to identify cell-type specific associations [4–8]. Thus, to avoid false-positive findings and to detect cell-type specific associations it is critical to know the proportions of the major cell-types in the investigated samples.

In transcriptome- and methylome-wide studies the cell-type proportions for each bulk sample are typically estimated from the data itself in combination with cell-type specific profiles from a reference panel of sorted cells from a small number of samples [9]. Unfortunately, in targeted studies where a limited number of sites/transcripts are investigated, the many cell-type markers typically used for estimating the proportions will not be assayed. Thus, correction for cell-type proportions in these studies are often overlooked, likely resulting in an increased number of false positive findings in targeted investigations such as replication studies. A solution would be to rely on cell-type proportions obtained from an independent approach, such as counts from flow cytometry. However, this adds significant costs and, particularly in studies involving previously collected and stored samples, technical limitations often make cell-sorting challenging or impossible to perform.

In this article we develop an alternative three-step approach that allows for estimation of cell-type proportions in targeted studies. First, we perform a cost-effective sequencing-based methylome-wide investigation assaying nearly all 28 million CpGs [10] to identify highly informative cell-type specific methylation markers. Next, we use a modified protocol for parallel bisulfite amplicon sequencing to create a reference panel by assaying the most informative methylation markers in sorted cells. Finally, we assay only the cell-type specific reference markers in the bulk samples to enable estimating the cell-type proportions in these samples. Further methodological details are provided in the Additional files 1 and 2. In theory this approach can be applied using any biomarker with cell-type specific profiles. However, we have chosen to use methylation markers as they are very stable over time in collected bio-samples.

## Results

We will illustrate the three-step approach with samples from human blood to estimate proportion of T-cells (CD3^+^ cells), monocytes (CD14^+^ cells), granulocytes (CD15^+^ cells), and B-cells (CD19^+^ cells). The methylome-wide investigation identified 53 loci that had nearly unique cell-type specific methylation profiles in all nine individuals investigated. Following stringent evaluation, high quality primer sets with on target amplification (Additional file 1: Figure S1) was achieved for 18 of these loci, including 37 assayed CpG sites (Additional file 2: Table S1). Using these 18 amplicons we created a reference panel, which was created of the four sorted blood cell-types from seven individuals. Next, we evaluated the cell-type estimates using five samples with known cell-type mixtures. Furthermore, we assayed five samples with known methylation levels and five bulk blood samples that were used to evaluate the methylation assessment protocol. Assays for all 15 samples were performed in duplicates.

Cell-type proportions, for the samples with known cell-type mixtures, were estimated with the commonly used ordinary least squares regression (OLS) approach as well as with weighted least squares regression as implemented in the MuSiC [11] package. Further details about these two approaches are provided in the methods section. Table 1 shows that the reliability of the approach in general was high. More specifically, the average correlations between the estimated and the true cell-type proportions where higher for MuSiC (r = 0.859–0.889) than for OLS (r = 0.768–0.784) indicating superior reliability of the estimates when using MuSiC as compared to the OLS approach. Furthermore, Table 1 shows that the most reliable estimates were obtained with 17 amplicons, after excluding one amplicon (P5, Additional file 2: Table S2). The estimates obtained with the optimized approach, i.e., the MuSiC estimator and 17 amplicons (34 CpGs), were not significantly different from the true values (*P* = 0.95) indicating that the estimator is unbiased. The standard deviation of the estimates further indicate high precision. Finally, the overall variability of the estimates as measured by the Root Mean Squared Error (RMSE) that is a function of both bias and precision was low (mean RMSE = 0.038). Taken together, these results indicate that the approach produced reliable estimates that are both unbiased and highly precise.

**Table 1:** Evaluation of cell-type proportion estimation in bulk samples with known cell type proportions

Method	MuSiC		OLS	
	*Number of included amplicons*			
Total	17	18	17	18
CD3	4	5	4	5
CD14	7	7	7	7
CD15	3	3	3	3
CD19	3	3	3	3
	*Correlation with true proportion*			
CD3	0.990	0.971	0.986	0.954
CD14	0.991	0.988	0.976	0.971
CD15	0.832	0.829	0.796	0.787
CD19	0.742	0.649	0.377	0.358
Mean	0.889	0.859	0.784	0.768
	*Mean cell-type proportion*			
CD3 (0.28)	0.30	0.27	0.30	0.28
CD14 (0.13)	0.14	0.14	0.16	0.17
CD15 (0.58)	0.52	0.53	0.50	0.51
CD19 (0.08)	0.05	0.05	0.04	0.04
	*Standard deviation of cell-type proportion*			
CD3 (0.20)	0.22	0.23	0.22	0.23
CD14 (0.03)	0.06	0.07	0.09	0.09
CD15 (0.20)	0.19	0.20	0.20	0.20
CD19 (0.03)	0.05	0.05	0.05	0.05
	*RMSE*			
CD3	0.042	0.057	0.047	0.067
CD14	0.042	0.048	0.073	0.081
CD15	0.026	0.029	0.049	0.047
CD19	0.040	0.040	0.055	0.057
Mean	0.038	0.044	0.056	0.063

The expected mean and SD are given in parenthesis

OLS is ordinary least square regression

**Fig. 1:**
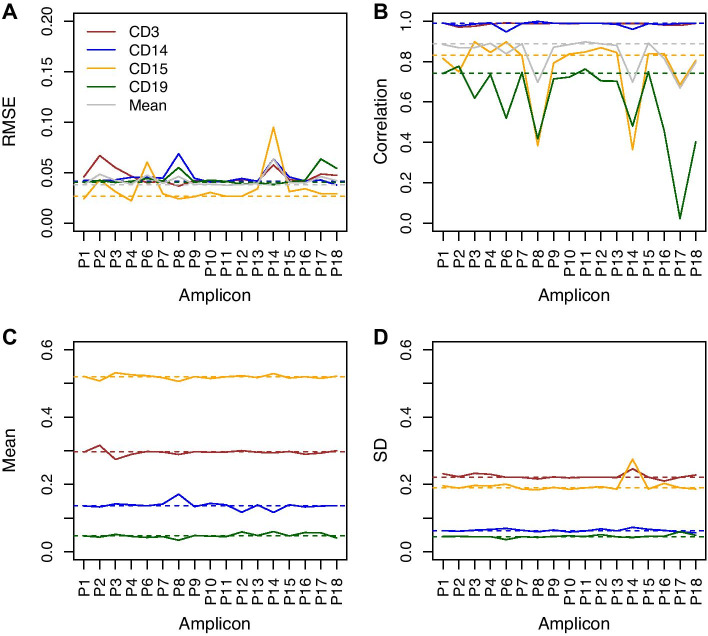
Performance of the cell-type estimation when a marker is missing. Performance of the cell-type estimation when an amplicon is excluded as compared to the optimal model. Solid lines indicate the performance of the estimation when an amplicon is excluded for CD3^+^, CD14^+^, CD15^+^ and CD19^+^, respectively. Dashed lines indicate the value of the optimal model. The excluded amplicon is indicated on the x-axis. The y-axes show **a** the root mean square error (RMSE); **b** the Pearson correlation between the estimated cell-type proportions and the true values; **c** the mean estimated cell-type proportion for the sample with known proportions; and **d** the standard deviation of the estimated cell-type proportions

To evaluate if the optimized approach is robust to potentially missing data we excluded one amplicon at a time and estimated the cell-type proportions. Figure 1 shows the results for each excluded marker as compared to the results from the optimal model described above (indicated as dashed lines in Fig. 1). The overall measures of variability (RMSE) and the specific measures for reliability (correlation), bias (mean) and precision (standard deviation) are affected to different degrees dependent on which marker is excluded. For example, if amplicon P14 or P17 are missing the overall variability increases and the mean correlation drastically decreases. Thus, we would suggest setting the cell-type proportions to missing for individuals with missing data for P14 or P17. On the contrary, lacking information from amplicons such as P9 or P12 will only have minor effect on the estimates of the cell-type proportions.

## Discussion

While the targeted parallel bisulfite amplicon sequencing protocol used in this study provides a cost-effective solution to obtain cell-type proportions, other methylation assays can be used. Moreover, the proposed 3-step approach of using a small number of carefully selected critical target sites to generate a reference panel for estimating cell-type proportions is applicable across tissues. Once the cell-type proportions have been estimated, they can be used to control for variation in cell-type proportions and to study cell-type-specific associations in any data, including both methylation data and transcription data, generated by any platform. Generated reference panels can potentially be made publicly available for a broad set of tissues, cell-types and species. Thus, in addition to directly serving the specific project it was generated for, these panel may also serve the broader research community as tissue/platform specific reference panels.

## Conclusion

In conclusion, the proposed approach allows for precise estimation of cell-type proportions using only a small number of amplicons. The same approach can be extended to assess cell-type proportions for any tissue.

## Methods

### Samples

#### Reference panel of sorted cell-types

For the methylome-wide investigation (N = 9) and for the targeted reference panel (N = 7) we used DNA extracted from sorted blood cells from US adult volunteer subjects that had been obtained from Virginia Blood Services. The different cell populations were isolated by positive selection using EasySep™ kits (Stemcell Technologies), which apply magnetic nanoparticles coated with antibodies against a particular surface antigen (CD molecules). Specifically, we isolated CD3^+^, CD14^+^, CD15^+^ and CD19^+^ cells corresponding to T-cells, monocytes, granulocytes, and B-cells, respectively.

#### Samples with known cell-type mixtures

In the lieu of bulk samples with known cell-type proportions we used genomic DNA from the sorted cells, from five individuals, to create bulk samples with known mixtures, in duplicates. The DNA from the sorted cells were pooled in predetermined proportions as indicated in Additional file 2: Table S3.

#### Samples with known methylation levels and bulk blood samples

We used fully (> 98%) CpG methylated human genomic DNA (cat# SD1131, Thermo Scientific) in combination with unmethylated DNA from the same individual to create samples with known methylation levels. First, an aliquot (400 ng) of the fully methylated DNA was used for whole genome amplification with the REPLI-g Mini kit (Qiagen) to create a corresponding non-methylated (0%) samples with the same genetic sequence. Next, the fully methylated and the non-methylated samples were pooled (Additional file 1: Figure S2) to create samples with 100%, 75%, 50%, 25% and 0% methylation levels.

In addition, we also included DNA extracted from buffy coat of whole blood, in duplicates, that are used to evaluate the methylation assessment for the included amplicons.

#### Blanks without DNA for quality control purposes

Finally, we included two “blanks”, which are identical to the samples with known methylation levels with the exception that DNA is excluded (i.e., replaced with the buffer in which the DNA is dissolved). Traditionally, the main purpose of blanks are to serve as negative controls to detect contamination. However, here they will also allow us to determine a background noise level for the sequencing depth required, which is critical for proper quality control.

### Methylome-wide investigation to identify cell-type specific methylation markers

Methylation sites with unique cell-type specific methylation profiles for four of the most common leucocyte types in blood (CD3^+^, CD14^+^, CD15^+^ and CD19^+^ cells corresponding to T-cells, monocytes, granulocytes, and B-cells, respectively) were identified using methylation profiles from the sorted blood cells from nine individuals. As previously described [12], the methylation profiles were generated using methyl-binding domain sequencing (MBD-seq) [7, 13], which allows for assessment of nearly all 28 million CpG sites in the human genome [10]. The methylation data was processed with the RaMWAS Bioconductor package [14]. RaMWAS quantifies methylation by estimating the number of fragments covering a CpG site using a non-parametric estimator of the fragment size distribution [15]. To identify cell-type specific makers, we calculated priority scores for each CpG by counting the number of samples with fragment coverage [15] higher than 0.3 for the target cell-type and fragment coverage lower than 0.3 for all other cell-types. Thus, the maximum priority score was 9 individuals × 4 cell-types = 36.

### Primer design and evaluation

For 53 loci that, according to the priority score described above, were determined to have unique methylation profiles for different cell-types, we designed primers for targeted amplicon bisulfite-sequencing using the Juno platform (Fluidigm). First, primers were designed for each site of interest using the Pyromark Assay Design 2.0 software (Qiagen). Second, platform-specific adapters were added and oligo properties such as melting temperature, hairpins, dimers, and genomic mismatches were evaluated using the OligoAnalyzer tool (Integrated DNA Technologies). Third, to detect non-specificity across the bisulfite-converted genome promising designs were further evaluated in silico via BiSearch [16, 17]. Finally, prior to using the primers on the Juno platform they were evaluated off the platform using the same cycling conditions (Additional file 2: Table S4) in 10ul PCR reactions, followed by evaluation of the amplification profile on a Bioanalyzer (Agilent).

### Assaying DNA methylation levels with targeted amplicon bisulfite sequencing

Genomic DNA was bisulfite converted using the EZ DNA Methylation-Lightning Kit (#D5030/D5032; company) followed by quantification with NanoDrop Spectrophotometer (ThermoFisher Scientific). A protocol for targeted assessment of DNA methylation using microfluidics has been developed previously [18]. Here we capitalize on this approach to develop a modified protocol for the Juno platform (Fluidigm) to generate targeted amplicon libraries for next-generation sequencing. For further details see the Additional files 1 and 2. Finally, the concentration of (pools of) libraries were measured with Qubit dsDNA HS Assay Kit (Thermo Fisher Scientific), pooled in equal molarities and sequenced with 75 bp single-end reads on a NextSeq500 platform (Illumina). The sequencing data was processed with BS-Seeker [19] using Bowtie2 [20] for alignment while allowing for one mismatch.

All amplicons were on target with an average per target read coverage of 2877 reads per sample (SD = 823). Across the entire genome, only five additional loci received a read coverage greater than 10, where the highest average coverage observed was 70. Thus, the amount of reads observed outside of the targeted loci were well below the reads observed on target and were even lower than the average number of reads observed on target for the blank controls (i.e., lower than the background noise level).

To call methylation levels for each investigated CpG (i.e., the percentage methylation) the number of observed reads with a cytosine at the targeted site was divided by the total number of reads at this location. If the number of reads for a specific amplicon, from a particular samples, did not exceed five times the background level (i.e., the average number of reads for this site observed for the blanks), the reads for this amplicon were excluded from further analysis.

### Estimating cell-type proportions

Cell-type proportions for each bulk sample were estimated with the help of a reference panel, that is generated with DNA from sorted cells, using either ordinary least squares regression (OLS) [9] or the weighted least squares regression (WLS) approach implemented in the R-packageMuSiC [11]. Although the estimations differ, both methods essentially use the same model. It is important to note that a separate regression analysis is performed for each subject. For each regression analysis, we regress the bulk methylation levels of each selected CpG on the mean methylation levels of the corresponding CpG in the reference panel. Intuitively speaking, the model assumes that the amount of methylation in bulk tissue is a weighted sum of the average methylation levels for each cell-type, with weights being equal to the cell-type proportion in bulk. However, as the methylation levels of these cell-types are unknown, the approach uses the mean methylation levels of the reference panel. The reference panel will not perfectly match the true cell-type methylation profiles for each subject and the model accounts for this by allowing residuals. Alternatively, the model can be explained by stating that it correlates the bulk methylation levels with the methylation levels of each cell-type in the reference panel. The higher the correlation with a specific cell-type, the more cells of that type is present in bulk. For example, if a reference cell-type would be perfectly correlated with bulk methylation levels, the model would infer that all cells are of that type. In contrast, if bulk methylation levels would be uncorrelated with a given reference cell-type, the model would infer that this cell-type is absent from the bulk tissue.

More formally we can write the model as:

$$Y_{i}^{bulk} = \sum\limits_{c = 1}^{{n_{c} }} {p_{i}^{c} R^{c} + E_{i} }$$

where the *m* × 1 vector $Y_{i}^{bulk}$ represents the bulk methylation measurements for subject *i* of the *m* selected CpGs, the *m* × 1 vector *R*^c^ contains the mean methylation of the *m* CpGs across the reference panel for cell-type *c*, and *E*_*i*_ is a *m* × 1 vector with residuals. The OLS estimator simply minimizes the sum of square differences between the observed and predicted values. Whereas in the OLS estimation each of the *m* CpGs have the same weight, in WLS some CpGs affect the estimation more than others. Specifically, MuSiC assigns higher weights to CpGs with consistent methylation levels across subjects (i.e., low cross-subject variance) and high variability across cell types (i.e., are more informative). In addition to using weights there are other estimation differences. For example, MuSiC employs a tree-guided procedure that recursively zooms in on closely related cell-types. This may contribute to the observation that MuSiC may outperform other methods [21, 22] especially when cell-types are closely related [11].

## Supplementary Information

**Additional file 1.** Supplementary Methods and Results.

**Additional file 2.** Supplementary Tables.

## Data Availability

The script used to estimate the cell-type proportions is made available via https://github.com/ejvandenoord/targeted_cell-type_estimation. We have made the methylation estimates and the data from the cell-type specific reference panel available (Additional file 2: Tables S5, S6).
