# Supplementary material for: A targeted solution for estimating the cell-type composition of bulk samples
Source: BMC Bioinformatics. 2021 Sep 26;22:462. doi: 10.1186/s12859-021-04385-0 (PMC8474864; doi:10.1186/s12859-021-04385-0)
Supplement: Supplementary file 1 — Additional file 1. Supplementary Methods and Results. [file 12859_2021_4385_MOESM1_ESM.pdf]

Supplemental Material

Table of Contents

Methods.. 2
Targeted amplicon sequencing with the Juno platform 2
Results.. 3
Targeted amplicon sequencing with the Juno platform 3
Figures 5
Figure S1. Read coverage of included amplicons.. 5
Figure S2. Mixing strategy to create DNA with known methylation levels............................... 6

Methods

Targeted amplicon sequencing with the Juno platform

According to the manufacturer of the Juno platform, Fluidigm, the optimal amount of input material for their standard targeted amplicon sequencing assay for non-bisulfite converted DNA is 100-200 ng DNA per reaction. However, for bisulfite converted DNA we used starting material in the upper area of this range. That is, 2ul of bisulfite converted DNA in a concentration of at least 100ng/ul was mixed with sample pre-mix solution and barcodes to create a Sample Plate. Furthermore, we prepared a Primer Assay Plate with 10X assay pools and assay pre-mix. This mix include forward and reverse primers to achieve a final concentration in the reaction chambers of 50nM and 16.7nM, respectively. The Sample Plate preparation, the Primer Assay Plate preparation as well as the loading and running of the Juno LP—192.24 IFC were performed according to the manufacturer's instructions. The original protocol suggests that all samples are pooled prior to further sample preparation. However, we achieved a more balanced distribution of the amplicon concentrations between different samples when the samples were harvested in pools of 8 (i.e., a total of 24 pools per 192.24 IFC). Next, for each pool, we performed three rounds of cleaning/size selection using Agencourt AMPure XP magnetic beads in a concentration of 1.6X/0.4X-0.9X of the sample volume. Following size selection, the amplicon profiles for each pool was evaluated with the high sensitivity DNA chip on the Bioanalyzer (Agilent). For pools with clean profiles, without indications of primer dimers, an adapter ligation PCR was performed to add sequencing adapters to the amplicons. Following the adapter ligation the PCR product for each pool was cleaned with 0.8X Agencourt AMPure XP magnetic beads to remove any access of adapters. The profile of the purified library pool was

again evaluated on the Bioanalyzer. To ensure that the adapters were successfully ligated onto the libraries, the Bioanalyzer profiles were compared with corresponding profiles from before adapter ligation to confirm that a ~30 bp size increase occurred. In the event that the library contained non-ligated adapters, an additional cleaning round with 1.0X Agencourt AMPure XP magnetic beads was performed. Finally, the concentration of the processed pools were measured with Qubit dsDNA HS Assay Kit (Thermo Fisher Scientific), pooled in equal molarities and sequenced with 75 bp single-end reads on a NextSeq500 platform (Illumina).

Results

Targeted amplicon sequencing with the Juno platform

The integrated fluidic circuits (IFC) on the Juno platform offers simultaneous amplification of 192 samples for 24 target loci (192.24 IFC). Therefore, as the present study involved analyses of 18 amplicons from 58 samples the remaining targets and sample slots were filled with miscellaneous probes and samples. In total, our assay generated methylation data of 190 bisulfite converted DNA samples that were amplified for 24 loci. **Figure S1** shows the read coverage for all amplicons used for estimating cell-type proportions. All amplicons were on target and were sequenced with an average per target read coverage of 2,877 reads per sample (SD = 823). For quality control purposes we added two blank controls (i.e., reactions where DNA was substituted for buffer), which received on average of 107 reads per sample (SD = 6). Thus, the background noise levels for the targets were low with a signal-to-noise ratio of 26.9. However, for one of the 24 primer sets the amplification failed for an unexpectedly large number of samples (N=23). This amplicon was therefore excluded from further analysis from all individuals. The remaining 23 amplicons included a total of 46 assayed CpG sites.

As explained in the online methods the 58 primary samples included a reference panel, Artificially created bulk samples with known cell-type mixtures, Bulk blood samples, Samples with known methylation levels. Across the 46 CpGs assayed for each of the 58 samples, 176 values (6.6%) did not pass our quality control threshold for read coverage and were set to missing. The majority (73%) of the missing values were caused by three samples for which amplification failed for nearly all sites, possibly due to a clog in the Juno integrated fluidic circuits (IFC), which may have prevented proper reagents/sample flow. After excluding all sites for these three samples, the missing rate among the remaining 55 samples, 28 of which were duplicate pairs, was reduced to 1.9%.

The average duplicate correlation for these samples, across the 46 investigated CpGs, was 0.96 (SD = 0.051). The methylation assessments were further evaluated by assessing the average correlation (r = 0.882, SD = 0.183) between the normalized methylation measures for the standards and the known theoretical methylation levels. The lower correlation observed here as compared to the duplicate correlation was mainly driven by missing values for two probes, six CpGs. Site specific duplicate correlations and the performance for the individual amplicons are shown in **Table S2**. As indicated in this table, 18 of these amplicons, covering 37 CpGs, were designed for estimating cell-type proportions and are the focus of the main analysis.

Figures

Figure S1. Read coverage of included amplicons.

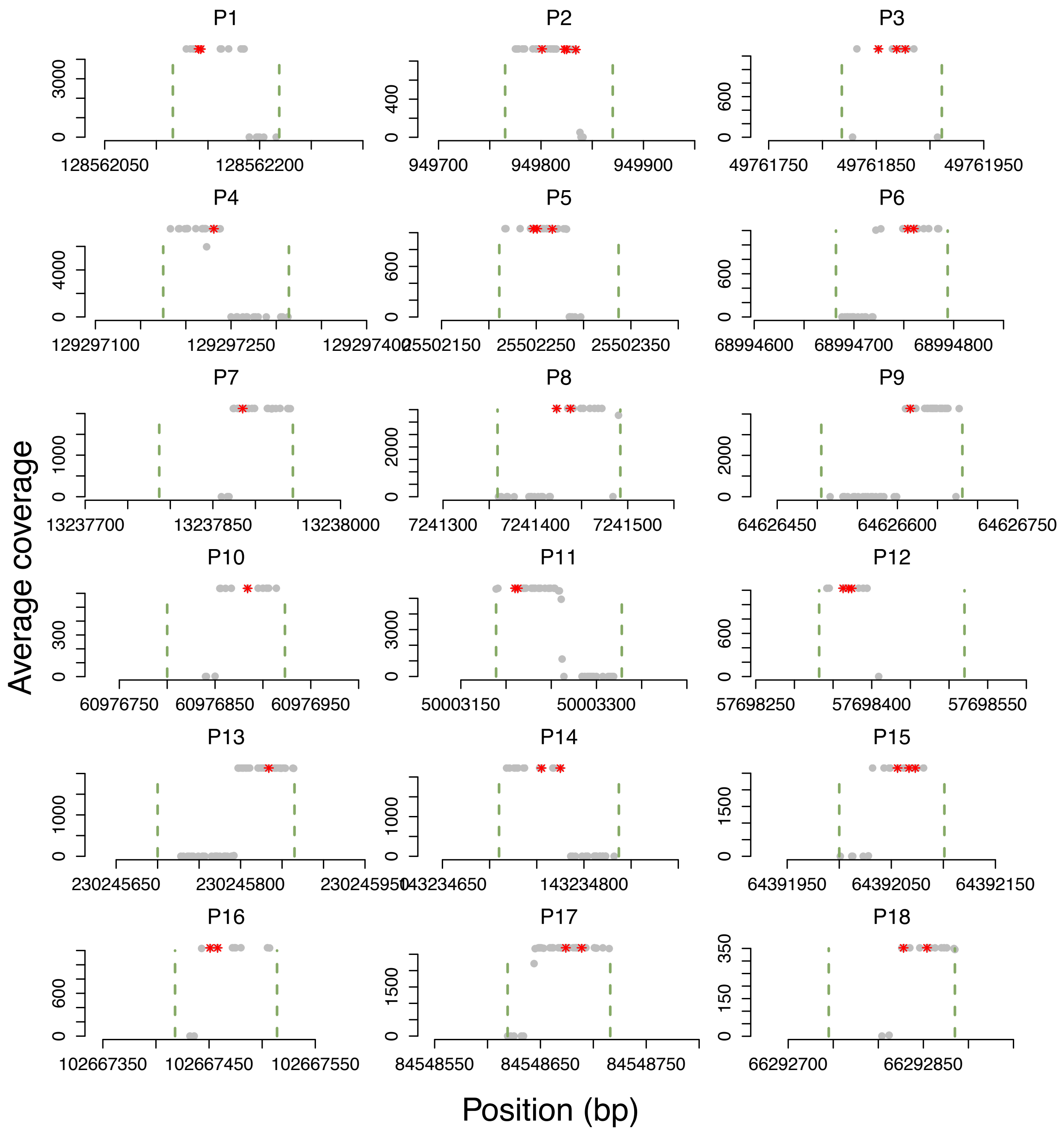

Plots for each of the 18 amplicons (indicated by green vertical bars) used to estimate cell-type proportions are shown. Specific details, linked via the P# identifier, can be found in Table S1. The X-axis indicates the genomic location in base pairs. The Y-axis indicate the average coverage across samples for all cytosines located within each amplicon. Red dots indicate CpG sites used for estimating cell type proportions. Note that up to 75 bp of each amplicon is sequenced. Thus, a sudden drop in read coverage is expected towards the end of the amplicon.

Figure S2. Mixing strategy to create DNA with known methylation levels.

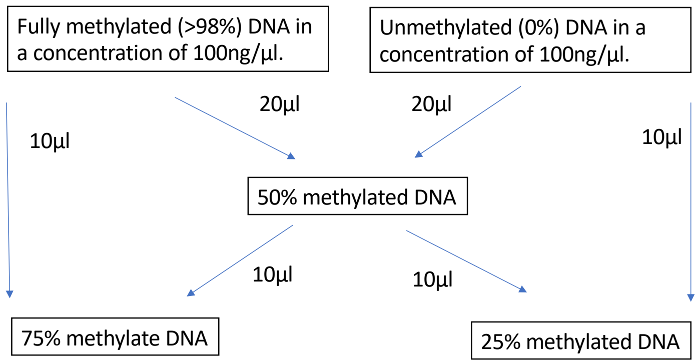

The mixing strategy for how samples with known methylation levels were created from the fully methylated and unmethylated samples. The amount of DNA that was transferred is indicated by the arrows.
